# 5,10,10,15,20,20-Hexamethylcalix[4]pyrrole 5,15-diethyl diester

**DOI:** 10.1107/S1600536809048260

**Published:** 2009-11-21

**Authors:** Abdullah Aydogan, Ahmet Akar

**Affiliations:** a Istanbul Technical University, Faculty of Science and Letters, Department of Chemistry, 34469 Maslak, Istanbul, Turkey

## Abstract

In the title compound, C_32_H_40_N_4_O_4_, the pyrrole rings and ester groups adopt a 1,3-alternate conformation in which the alternating pyrrole and ester units are in opposite directions. The structure displays N—H⋯O hydrogen bonding and exhibits disorder [site occupancies of 0.81(2) and 0.71(2)] in the peripheral ethyl groups.

## Related literature

For related calix[4]pyrrole structures see: Gale *et al.* (1998 ▶, 2001 ▶). For the synthesis of mono- and di-ester functionalized calix[4]pyrrole structures, see: Akar & Aydogan (2005 ▶). For applications of calix[4]pyrroles, see: Varo *et al.* (1996 ▶); Beer & Gale (2001 ▶); Nishiyabu & Pavel Anzenbacher (2005 ▶); Miyaji *et al.* (1999 ▶); Nielsen *et al.* (2004 ▶); Sessler *et al.* (1998 ▶).
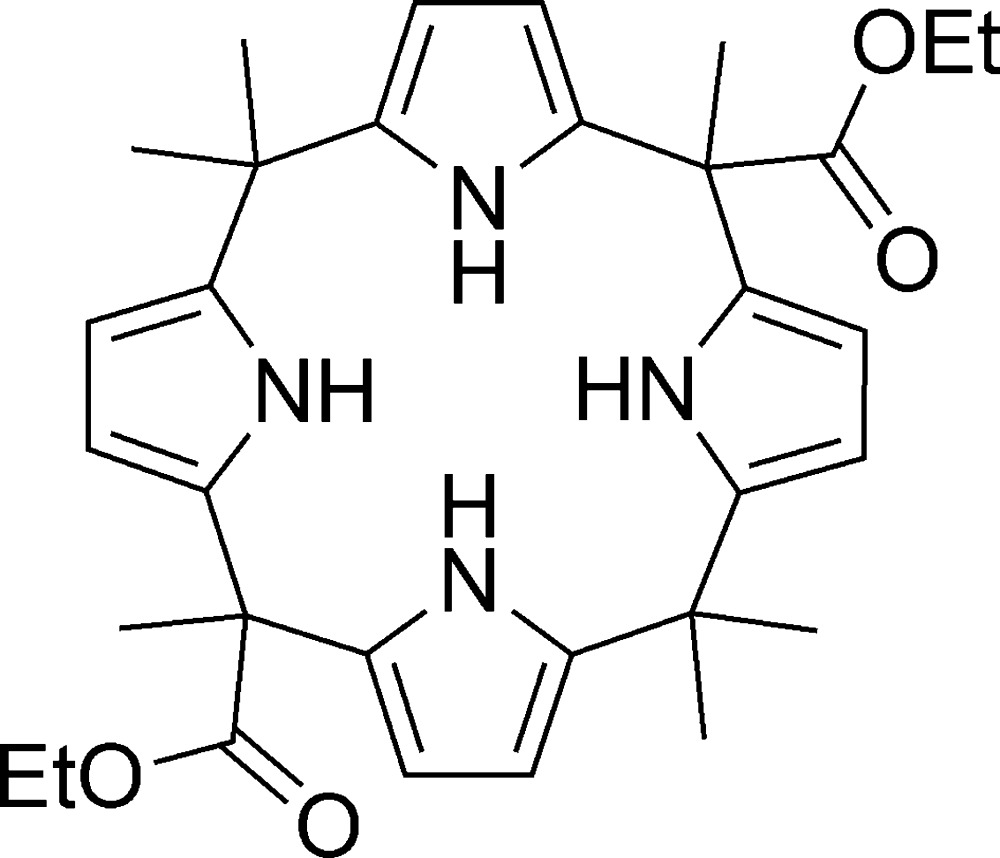


## Experimental

### 

#### Crystal data

C_32_H_40_N_4_O_4_
*M* *_r_* = 544.68
Monoclinic, 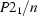

*a* = 10.4392 (3) Å
*b* = 11.6453 (3) Å
*c* = 24.0488 (7) Å
β = 95.380 (2)°
*V* = 2910.68 (14) Å^3^
*Z* = 4
Mo *K*α radiation
μ = 0.08 mm^−1^
*T* = 153 K
0.30 × 0.30 × 0.23 mm

#### Data collection

Nonius KappaCCD diffractometer
Absorption correction: none
11684 measured reflections
6619 independent reflections
2971 reflections with *I* > 2σ(*I*)
*R*_int_ = 0.064

#### Refinement

*R*[*F* ^2^ > 2σ(*F* ^2^)] = 0.057
*wR*(*F* ^2^) = 0.135
*S* = 1.09
6619 reflections
390 parameters
12 restraints
H atoms treated by a mixture of independent and constrained refinement
Δρ_max_ = 0.26 e Å^−3^
Δρ_min_ = −0.30 e Å^−3^

### 

Data collection: *COLLECT* (Nonius, 1998 ▶); cell refinement: *COLLECT*; data reduction: *DENZO* and *SCALEPACK* (Otwinowski & Minor, 1997 ▶); program(s) used to solve structure: *SIR97* (Altomare *et al.*, 1999 ▶); program(s) used to refine structure: *SHELXTL/PC* (Sheldrick, 2008 ▶); molecular graphics: *SHELXTL/PC*; software used to prepare material for publication: *SHELXTL/PC*.

## Supplementary Material

Crystal structure: contains datablocks I, global. DOI: 10.1107/S1600536809048260/om2288sup1.cif

Structure factors: contains datablocks I. DOI: 10.1107/S1600536809048260/om2288Isup2.hkl

Additional supplementary materials:  crystallographic information; 3D view; checkCIF report

## Figures and Tables

**Table 1:** Hydrogen-bond geometry (Å, °)

*D*—H⋯*A*	*D*—H	H⋯*A*	*D*⋯*A*	*D*—H⋯*A*
N2—H2*N*⋯O25^i^	0.86 (2)	2.37 (2)	3.211 (3)	167 (2)

Symmetry code: (i) 
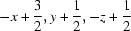
.

**Fig. 1.**
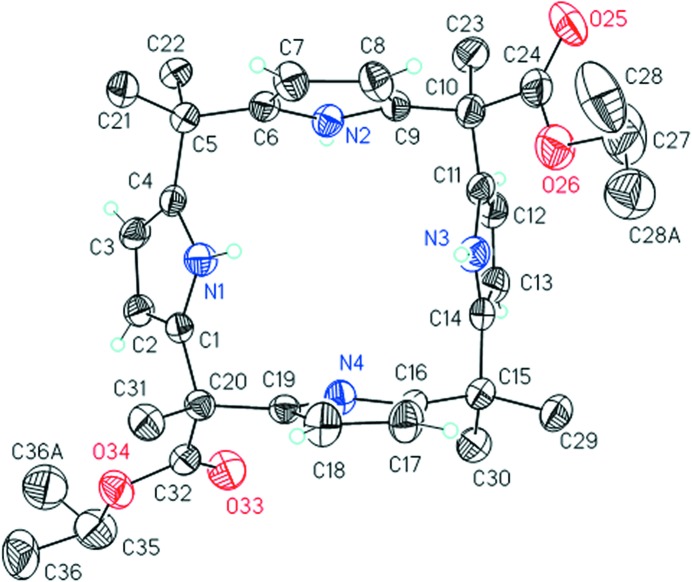
View of the title compound showing the atom labeling scheme. Displacement ellipsoids are scaled to the 50% probability level. Most hydrogen atoms have been removed for clarity. The methyl groups on both esters were disordered about two positions as shown. The minor occupancy groups have labels appended by A.

**Fig. 2.**
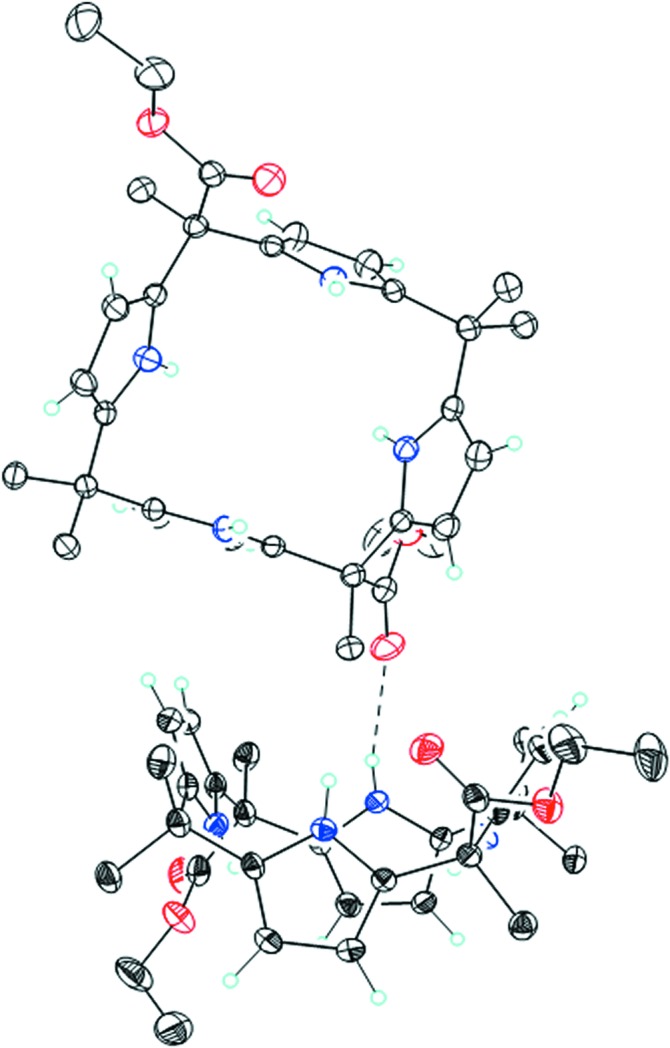
View illustrating a portion of the H-bonding interactions between molecules along the twofold screw axis. Displacement ellipsoids are scaled to the 30% probability level. Most hydrogen atoms have been removed for clarity.

**Fig. 3.**
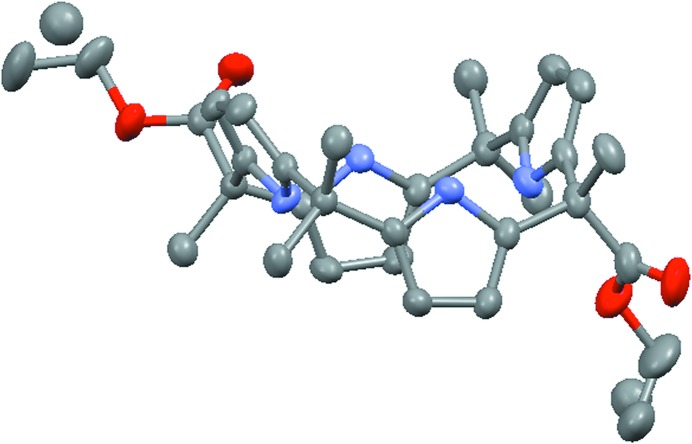
View of the 1,3-alternate conformation of the title compound. Neighboring pyrrole rings and ester groups are oriented in opposite directions. Hydrogen atoms have been removed for clarity.

## supplementary crystallographic information

### Comment

Anion receptors have become a focus of the research field of supramolecular chemistry because of the important roles of anions in biomedicine (Varo *et al.*, 1996) and enviromental processes (Beer & Gale, 2001). In addition anion receptors can be used as ion-selective receptors (Nishiyabu & Pavel Anzenbacher, 2005), phase-transfer catalysts (Miyaji *et al.*, 1999), ion-selective optical sensors (Nielsen *et al.*, 2004) and chromatographic separation systems (Sessler *et al.*, 1998).

In this context, calix[4]pyrroles have emerged as molecules of particular interest because of their simple preparations in one-step and easy modification of their core structures.

The title compound is shown in Fig. 1. It exhibits a strong intermolecular H-bonding interaction as depicted in Fig. 2.

As it can be seen in Fig. 3, pyrrole units of title compound adopt 1,3-alternate conformation which is the nitrogen atoms of neighboring pyrroles oriented in opposite directions. It is also observed that the ester groups are in opposite directions according to calixpyrrole plane and *meso*-carbon atoms containing ester groups are connected to different pyrrole rings.

### Experimental

Synthesis of the title compound was carried out according to a previously reported procedure (Akar & Aydogan, 2005).

The sample grew as very large, yellow prisms by slow evaporation from methylene chloride/diethylether. The data crystal was cut from a large specimen.

### Refinement

The hydrogen atoms on carbon were calculated in ideal positions with isotropic displacement parameters set to 1.2 x Ueq of the attached atom (1.5 x Ueq for methyl hydrogen atoms). The hydrogen atoms on the pyrrole nitrogen atoms were observed in a difference Fourier map and refined with isotropic displacement parameters. Both methyl groups on the ester moieties were disordered about two orientations. The disorder was modeled in the same way for both groups. The site occupancy for one carbon atom orientation was assigned a variable *x*. The site occupancy factor for the other conformer was assigned the variable (1 - *x*). The variable *x* was refined while refining the two atoms with a single isotropic displacement parameter. At the same time, the geometry of the methyl carbon atoms were restrained to be equivalent. In this way, the site occupancy factor for C36 refined to 81 (2)% and that for C28 refined to 77 (2)%. The lower occupancy carbon atoms, C28A and C36A, were refined isotropically.

### Crystal data

**Table:**

C_32_H_40_N_4_O_4_	*F*(000) = 1168
*M**_r_* = 544.68	*D*_x_ = 1.243 Mg m^−^^3^
Monoclinic, *P*2_1_/*n*	Mo *K*α radiation, λ = 0.71073 Å
*a* = 10.4392 (3) Å	Cell parameters from 5852 reflections
*b* = 11.6453 (3) Å	θ = 2.9–27.5°
*c* = 24.0488 (7) Å	µ = 0.08 mm^−^^1^
β = 95.380 (2)°	*T* = 153 K
*V* = 2910.68 (14) Å^3^	Prisms, yellow
*Z* = 4	0.30 × 0.30 × 0.23 mm

### Data collection

**Table:**

Nonius KappaCCD diffractometer	2971 reflections with *I* > 2σ(*I*)
Radiation source: fine-focus sealed tube	*R*_int_ = 0.064
graphite	θ_max_ = 27.5°, θ_min_ = 3.0°
ω scans	*h* = −13→13
11684 measured reflections	*k* = −15→15
6619 independent reflections	*l* = −31→31

### Refinement

**Table:**

Refinement on *F*^2^	Secondary atom site location: difference Fourier map
Least-squares matrix: full	Hydrogen site location: inferred from neighbouring sites
*R*[*F*^2^ > 2σ(*F*^2^)] = 0.057	H atoms treated by a mixture of independent and constrained refinement
*wR*(*F*^2^) = 0.135	*w* = 1/[σ^2^(*F*_o_^2^) + (0.044*P*)^2^] where *P* = (*F*_o_^2^ + 2*F*_c_^2^)/3
*S* = 1.09	(Δ/σ)_max_ = 0.035
6619 reflections	Δρ_max_ = 0.26 e Å^−^^3^
390 parameters	Δρ_min_ = −0.30 e Å^−^^3^
12 restraints	Extinction correction: *SHELXTL/PC* (Sheldrick, 1998), Fc^*^=kFc[1+0.001xFc^2^λ^3^/sin(2θ)]^-1/4^
Primary atom site location: structure-invariant direct methods	Extinction coefficient: 0.0023 (5)

### Special details

**Table:**

Geometry. All e.s.d.'s (except the e.s.d. in the dihedral angle between two l.s. planes) are estimated using the full covariance matrix. The cell e.s.d.'s are taken into account individually in the estimation of e.s.d.'s in distances, angles and torsion angles; correlations between e.s.d.'s in cell parameters are only used when they are defined by crystal symmetry. An approximate (isotropic) treatment of cell e.s.d.'s is used for estimating e.s.d.'s involving l.s. planes.
Refinement. Refinement of *F*^2^ against ALL reflections. The weighted *R*-factor *wR* and goodness of fit *S* are based on *F*^2^, conventional *R*-factors *R* are based on *F*, with *F* set to zero for negative *F*^2^. The threshold expression of *F*^2^ > σ(*F*^2^) is used only for calculating *R*-factors(gt) *etc*. and is not relevant to the choice of reflections for refinement. *R*-factors based on *F*^2^ are statistically about twice as large as those based on *F*, and *R*- factors based on ALL data will be even larger.Both methyl groups on the ester moieties were disordered about two orientations. The disorder was modeled in the same way for both groups. The site occupancy for one carbon atom orientation was assigned a variable *x*. The site occupancy factor for the other conformer was assigned the variable (1 - *x*). The variable *x* was refined while refining the two atoms with a single isotropic displacement parameter. At the same time, the geometry of the methyl carbon atoms were restrained to be equivalent. In this way, the site occupancy factor for C36 refined to 81 (2)% and that for C28 refined to 77 (2)%. The lower occupancy carbon atoms, C28A and C36A, were refined isotropically.

### Fractional atomic coordinates and isotropic or equivalent isotropic displacement parameters (Å^2^)

**Table:**

	*x*	*y*	*z*	*U*_iso_*/*U*_eq_	Occ. (<1)
N1	0.41232 (19)	0.58854 (17)	0.12330 (8)	0.0335 (5)	
N2	0.57872 (18)	0.40469 (18)	0.21083 (8)	0.0320 (5)	
N3	0.86084 (19)	0.48641 (18)	0.15438 (9)	0.0364 (5)	
N4	0.69557 (17)	0.70790 (17)	0.08407 (9)	0.0332 (5)	
C1	0.41029 (19)	0.70290 (18)	0.10945 (9)	0.0294 (6)	
C2	0.3522 (2)	0.7587 (2)	0.14962 (9)	0.0353 (6)	
H2	0.3358	0.8397	0.1507	0.042*	
C3	0.3188 (2)	0.6765 (2)	0.18906 (10)	0.0351 (6)	
H3	0.2777	0.6920	0.2224	0.042*	
C4	0.35692 (19)	0.57116 (19)	0.17249 (9)	0.0289 (6)	
C5	0.3415 (2)	0.45208 (18)	0.19602 (9)	0.0296 (6)	
C6	0.4570 (2)	0.38006 (19)	0.18602 (9)	0.0302 (6)	
C7	0.4680 (2)	0.2850 (2)	0.15386 (10)	0.0373 (6)	
H7	0.3987	0.2475	0.1319	0.045*	
C8	0.5988 (2)	0.2511 (2)	0.15865 (10)	0.0394 (6)	
H8	0.6346	0.1872	0.1401	0.047*	
C9	0.6660 (2)	0.32472 (19)	0.19419 (9)	0.0312 (6)	
C10	0.8051 (2)	0.3271 (2)	0.21892 (10)	0.0372 (6)	
C11	0.8675 (2)	0.4401 (2)	0.20719 (10)	0.0343 (6)	
C12	0.9319 (2)	0.5184 (2)	0.24153 (10)	0.0398 (6)	
H12	0.9505	0.5087	0.2811	0.048*	
C13	0.9654 (2)	0.6129 (2)	0.20934 (10)	0.0395 (6)	
H13	1.0120	0.6799	0.2229	0.047*	
C14	0.9206 (2)	0.5919 (2)	0.15496 (10)	0.0324 (6)	
C15	0.9303 (2)	0.65566 (19)	0.10072 (10)	0.0357 (6)	
C16	0.8010 (2)	0.65376 (19)	0.06609 (10)	0.0314 (6)	
C17	0.7601 (2)	0.6029 (2)	0.01682 (10)	0.0412 (7)	
H17	0.8129	0.5597	−0.0062	0.049*	
C18	0.6277 (2)	0.6277 (2)	0.00454 (10)	0.0412 (6)	
H18	0.5728	0.6012	−0.0272	0.049*	
C19	0.5895 (2)	0.69349 (18)	0.04663 (9)	0.0282 (5)	
C20	0.4601 (2)	0.74499 (18)	0.05557 (9)	0.0290 (6)	
C21	0.2204 (2)	0.39553 (19)	0.16701 (10)	0.0388 (6)	
H21A	0.2292	0.3889	0.1278	0.058*	
H21B	0.2085	0.3206	0.1824	0.058*	
H21C	0.1472	0.4427	0.1725	0.058*	
C22	0.3263 (2)	0.46188 (19)	0.25879 (9)	0.0380 (6)	
H22A	0.4022	0.4963	0.2774	0.057*	
H22B	0.2529	0.5089	0.2642	0.057*	
H22C	0.3143	0.3869	0.2740	0.057*	
C23	0.8124 (2)	0.3064 (2)	0.28177 (10)	0.0464 (7)	
H23A	0.7728	0.3686	0.3000	0.070*	
H23B	0.7676	0.2362	0.2881	0.070*	
H23C	0.9006	0.2994	0.2967	0.070*	
C24	0.8709 (2)	0.2257 (2)	0.19279 (11)	0.0450 (7)	
O25	0.89367 (17)	0.13441 (17)	0.21515 (8)	0.0633 (6)	
O26	0.89694 (16)	0.24818 (15)	0.14039 (7)	0.0576 (5)	
C27	0.9610 (3)	0.1595 (3)	0.11130 (14)	0.0835 (11)	
H27C	1.0493	0.1554	0.1263	0.100*	0.23
H27D	0.9216	0.0867	0.1176	0.100*	0.23
H27A	1.0173	0.1177	0.1379	0.100*	0.77
H27B	1.0123	0.1939	0.0847	0.100*	0.77
C28	0.8770 (4)	0.0833 (3)	0.08351 (19)	0.0866 (14)	0.77
H28A	0.9235	0.0259	0.0650	0.130*	0.77
H28B	0.8272	0.0472	0.1102	0.130*	0.77
H28C	0.8206	0.1239	0.0564	0.130*	0.77
C28A	0.9568 (12)	0.1779 (10)	0.0542 (3)	0.065 (4)*	0.23
H28D	1.0006	0.1173	0.0366	0.098*	0.23
H28E	0.8686	0.1806	0.0388	0.098*	0.23
H28F	0.9976	0.2499	0.0476	0.098*	0.23
C29	1.0315 (2)	0.5981 (2)	0.06754 (10)	0.0445 (7)	
H29A	1.0052	0.5207	0.0588	0.067*	
H29B	1.0404	0.6396	0.0336	0.067*	
H29C	1.1126	0.5973	0.0900	0.067*	
C30	0.9734 (2)	0.7806 (2)	0.11352 (11)	0.0475 (7)	
H30A	0.9107	0.8184	0.1338	0.071*	
H30B	1.0547	0.7791	0.1358	0.071*	
H30C	0.9825	0.8214	0.0794	0.071*	
C31	0.3626 (2)	0.7138 (2)	0.00625 (9)	0.0401 (6)	
H31A	0.3936	0.7393	−0.0280	0.060*	
H31B	0.3514	0.6320	0.0051	0.060*	
H31C	0.2817	0.7500	0.0108	0.060*	
C32	0.4738 (2)	0.8754 (2)	0.06379 (10)	0.0350 (6)	
O33	0.56342 (17)	0.92236 (14)	0.08880 (7)	0.0483 (5)	
O34	0.36920 (15)	0.93043 (13)	0.04203 (7)	0.0469 (5)	
C35	0.3633 (3)	1.0521 (2)	0.05480 (13)	0.0615 (9)	
H35C	0.4456	1.0784	0.0710	0.074*	0.19
H35D	0.3400	1.0956	0.0214	0.074*	0.19
H35A	0.4335	1.0915	0.0401	0.074*	0.81
H35B	0.3700	1.0626	0.0946	0.074*	0.81
C36	0.2440 (3)	1.0986 (3)	0.03141 (18)	0.0751 (13)	0.81
H36A	0.2399	1.1790	0.0399	0.113*	0.81
H36B	0.2374	1.0882	−0.0084	0.113*	0.81
H36C	0.1744	1.0593	0.0466	0.113*	0.81
C36A	0.2700 (13)	1.0685 (10)	0.0931 (6)	0.072 (5)*	0.19
H36D	0.2637	1.1482	0.1026	0.109*	0.19
H36E	0.1882	1.0420	0.0764	0.109*	0.19
H36F	0.2944	1.0247	0.1262	0.109*	0.19
H1N	0.453 (2)	0.5350 (18)	0.1093 (9)	0.031 (7)*	
H2N	0.600 (2)	0.4638 (19)	0.2312 (9)	0.040 (8)*	
H3N	0.819 (2)	0.4537 (18)	0.1267 (9)	0.029 (7)*	
H4N	0.698 (2)	0.752 (2)	0.1135 (10)	0.053 (8)*	

### Atomic displacement parameters (Å^2^)

**Table:**

	*U*^11^	*U*^22^	*U*^33^	*U*^12^	*U*^13^	*U*^23^
N1	0.0380 (12)	0.0285 (13)	0.0356 (13)	0.0067 (10)	0.0116 (10)	−0.0012 (10)
N2	0.0303 (12)	0.0319 (13)	0.0334 (12)	−0.0028 (10)	0.0021 (10)	−0.0029 (10)
N3	0.0301 (12)	0.0465 (14)	0.0320 (14)	−0.0011 (11)	−0.0003 (10)	0.0010 (12)
N4	0.0322 (13)	0.0405 (13)	0.0268 (12)	0.0038 (10)	0.0030 (10)	−0.0034 (11)
C1	0.0247 (12)	0.0273 (14)	0.0361 (15)	0.0016 (10)	0.0027 (11)	0.0029 (12)
C2	0.0340 (14)	0.0289 (13)	0.0448 (16)	0.0060 (11)	0.0126 (12)	0.0005 (13)
C3	0.0334 (14)	0.0359 (15)	0.0383 (15)	0.0029 (12)	0.0144 (12)	0.0002 (12)
C4	0.0222 (12)	0.0340 (14)	0.0310 (14)	0.0021 (11)	0.0047 (11)	0.0008 (12)
C5	0.0262 (13)	0.0306 (14)	0.0322 (14)	−0.0004 (11)	0.0046 (11)	0.0013 (11)
C6	0.0269 (14)	0.0312 (14)	0.0319 (14)	−0.0026 (11)	−0.0001 (11)	0.0037 (12)
C7	0.0354 (15)	0.0376 (15)	0.0374 (15)	0.0017 (12)	−0.0038 (12)	−0.0049 (13)
C8	0.0394 (15)	0.0387 (15)	0.0398 (16)	0.0105 (13)	0.0030 (12)	−0.0015 (13)
C9	0.0293 (14)	0.0355 (14)	0.0294 (14)	0.0053 (12)	0.0058 (11)	0.0063 (12)
C10	0.0292 (14)	0.0507 (16)	0.0324 (15)	0.0067 (12)	0.0064 (11)	0.0080 (13)
C11	0.0253 (13)	0.0509 (16)	0.0270 (15)	0.0044 (12)	0.0046 (11)	0.0087 (13)
C12	0.0339 (15)	0.0558 (17)	0.0296 (15)	0.0063 (13)	0.0021 (12)	0.0009 (14)
C13	0.0327 (14)	0.0479 (17)	0.0379 (16)	−0.0016 (12)	0.0030 (12)	−0.0064 (14)
C14	0.0219 (13)	0.0361 (15)	0.0396 (16)	0.0045 (11)	0.0045 (11)	0.0032 (12)
C15	0.0295 (14)	0.0411 (15)	0.0375 (15)	0.0029 (11)	0.0093 (12)	0.0058 (13)
C16	0.0275 (13)	0.0365 (14)	0.0314 (15)	0.0033 (11)	0.0097 (12)	0.0049 (12)
C17	0.0393 (16)	0.0466 (16)	0.0389 (16)	0.0074 (13)	0.0094 (13)	−0.0089 (13)
C18	0.0388 (16)	0.0494 (16)	0.0345 (15)	0.0012 (13)	−0.0014 (12)	−0.0119 (13)
C19	0.0305 (13)	0.0290 (13)	0.0254 (13)	0.0001 (11)	0.0037 (11)	0.0025 (11)
C20	0.0301 (13)	0.0274 (13)	0.0298 (14)	0.0013 (11)	0.0034 (11)	0.0005 (11)
C21	0.0287 (14)	0.0377 (15)	0.0497 (16)	−0.0001 (11)	0.0014 (12)	0.0029 (12)
C22	0.0345 (14)	0.0382 (15)	0.0425 (16)	−0.0006 (12)	0.0100 (12)	0.0067 (12)
C23	0.0375 (15)	0.0659 (18)	0.0349 (15)	−0.0033 (13)	−0.0011 (12)	0.0147 (14)
C24	0.0305 (15)	0.0565 (19)	0.0478 (18)	0.0063 (13)	0.0030 (13)	0.0123 (16)
O25	0.0570 (13)	0.0590 (13)	0.0736 (15)	0.0257 (10)	0.0044 (10)	0.0218 (11)
O26	0.0627 (12)	0.0598 (12)	0.0536 (13)	0.0199 (10)	0.0230 (10)	0.0037 (10)
C27	0.089 (3)	0.082 (3)	0.082 (3)	0.036 (2)	0.026 (2)	−0.015 (2)
C28	0.087 (3)	0.070 (3)	0.098 (4)	0.034 (3)	−0.015 (3)	−0.025 (3)
C29	0.0347 (15)	0.0597 (18)	0.0408 (16)	0.0062 (13)	0.0123 (12)	0.0075 (14)
C30	0.0375 (15)	0.0481 (17)	0.0568 (18)	−0.0038 (13)	0.0043 (13)	0.0081 (14)
C31	0.0380 (15)	0.0440 (15)	0.0379 (16)	0.0027 (12)	0.0005 (12)	0.0045 (13)
C32	0.0332 (15)	0.0375 (15)	0.0352 (15)	0.0027 (13)	0.0084 (12)	0.0042 (13)
O33	0.0475 (11)	0.0368 (10)	0.0592 (13)	−0.0056 (9)	−0.0024 (9)	−0.0044 (9)
O34	0.0428 (11)	0.0307 (10)	0.0671 (13)	0.0075 (8)	0.0047 (9)	0.0049 (9)
C35	0.069 (2)	0.0301 (16)	0.088 (2)	0.0102 (14)	0.0207 (18)	0.0045 (15)
C36	0.051 (2)	0.045 (2)	0.129 (4)	0.0151 (19)	0.010 (2)	0.008 (2)

### Geometric parameters (Å, °)

**Table:**

N1—C1	1.372 (3)	C21—H21A	0.9601
N1—C4	1.379 (3)	C21—H21B	0.9600
N1—H1N	0.84 (2)	C21—H21C	0.9600
N2—C6	1.382 (3)	C22—H22A	0.9600
N2—C9	1.387 (3)	C22—H22B	0.9600
N2—H2N	0.86 (2)	C22—H22C	0.9600
N3—C11	1.376 (3)	C23—H23A	0.9600
N3—C14	1.377 (3)	C23—H23B	0.9600
N3—H3N	0.85 (2)	C23—H23C	0.9600
N4—C19	1.370 (3)	C24—O25	1.205 (3)
N4—C16	1.373 (3)	C24—O26	1.340 (3)
N4—H4N	0.87 (2)	O26—C27	1.446 (3)
C1—C2	1.354 (3)	C27—C28	1.375 (4)
C1—C20	1.522 (3)	C27—C28A	1.387 (7)
C2—C3	1.414 (3)	C27—H27C	0.9599
C2—H2	0.9601	C27—H27D	0.9600
C3—C4	1.360 (3)	C27—H27A	0.9600
C3—H3	0.9602	C27—H27B	0.9601
C4—C5	1.512 (3)	C28—H28A	0.9600
C5—C6	1.507 (3)	C28—H28B	0.9601
C5—C21	1.534 (3)	C28—H28C	0.9600
C5—C22	1.537 (3)	C28A—H28D	0.9601
C6—C7	1.361 (3)	C28A—H28E	0.9600
C7—C8	1.417 (3)	C28A—H28F	0.9600
C7—H7	0.9600	C29—H29A	0.9600
C8—C9	1.358 (3)	C29—H29B	0.9597
C8—H8	0.9600	C29—H29C	0.9602
C9—C10	1.518 (3)	C30—H30A	0.9600
C10—C11	1.506 (3)	C30—H30B	0.9600
C10—C23	1.526 (3)	C30—H30C	0.9600
C10—C24	1.531 (3)	C31—H31A	0.9603
C11—C12	1.364 (3)	C31—H31B	0.9600
C12—C13	1.409 (3)	C31—H31C	0.9599
C12—H12	0.9601	C32—O33	1.196 (3)
C13—C14	1.369 (3)	C32—O34	1.330 (3)
C13—H13	0.9600	O34—C35	1.453 (3)
C14—C15	1.513 (3)	C35—C36A	1.414 (7)
C15—C16	1.518 (3)	C35—C36	1.424 (4)
C15—C29	1.537 (3)	C35—H35C	0.9600
C15—C30	1.545 (3)	C35—H35D	0.9599
C16—C17	1.358 (3)	C35—H35A	0.9599
C17—C18	1.416 (3)	C35—H35B	0.9604
C17—H17	0.9598	C36—H36A	0.9601
C18—C19	1.359 (3)	C36—H36B	0.9600
C18—H18	0.9599	C36—H36C	0.9600
C19—C20	1.512 (3)	C36A—H36D	0.9600
C20—C31	1.532 (3)	C36A—H36E	0.9601
C20—C32	1.536 (3)	C36A—H36F	0.9601
			
C1—N1—C4	110.63 (19)	C5—C22—H22A	109.3
C1—N1—H1N	128.3 (14)	C5—C22—H22B	109.4
C4—N1—H1N	120.1 (15)	H22A—C22—H22B	109.5
C6—N2—C9	109.7 (2)	C5—C22—H22C	109.7
C6—N2—H2N	125.8 (15)	H22A—C22—H22C	109.5
C9—N2—H2N	124.2 (15)	H22B—C22—H22C	109.5
C11—N3—C14	110.9 (2)	C10—C23—H23A	110.4
C11—N3—H3N	121.6 (15)	C10—C23—H23B	108.2
C14—N3—H3N	127.3 (15)	H23A—C23—H23B	109.5
C19—N4—C16	111.1 (2)	C10—C23—H23C	109.8
C19—N4—H4N	124.6 (16)	H23A—C23—H23C	109.5
C16—N4—H4N	123.9 (16)	H23B—C23—H23C	109.5
C2—C1—N1	106.9 (2)	O25—C24—O26	122.9 (2)
C2—C1—C20	131.7 (2)	O25—C24—C10	125.1 (2)
N1—C1—C20	121.36 (19)	O26—C24—C10	112.0 (2)
C1—C2—C3	108.0 (2)	C24—O26—C27	117.3 (2)
C1—C2—H2	125.7	C28—C27—O26	113.2 (3)
C3—C2—H2	126.3	C28A—C27—O26	113.3 (5)
C4—C3—C2	108.44 (19)	C28A—C27—H27C	108.7
C4—C3—H3	125.5	O26—C27—H27C	108.9
C2—C3—H3	126.1	C28A—C27—H27D	108.6
C3—C4—N1	106.06 (19)	O26—C27—H27D	109.3
C3—C4—C5	132.3 (2)	H27C—C27—H27D	107.9
N1—C4—C5	121.57 (19)	C28—C27—H27A	108.7
C6—C5—C4	109.62 (17)	O26—C27—H27A	108.7
C6—C5—C21	109.06 (18)	C28—C27—H27B	108.4
C4—C5—C21	109.60 (18)	O26—C27—H27B	109.6
C6—C5—C22	110.87 (18)	H27A—C27—H27B	108.1
C4—C5—C22	108.90 (18)	C27—C28—H28A	110.4
C21—C5—C22	108.77 (17)	C27—C28—H28B	108.5
C7—C6—N2	106.9 (2)	H28A—C28—H28B	109.5
C7—C6—C5	130.9 (2)	C27—C28—H28C	109.5
N2—C6—C5	122.2 (2)	H28A—C28—H28C	109.5
C6—C7—C8	108.2 (2)	H28B—C28—H28C	109.5
C6—C7—H7	125.7	H28A—C28—H28E	100.7
C8—C7—H7	126.1	C27—C28A—H28D	110.5
C9—C8—C7	108.2 (2)	H28C—C28A—H28D	101.1
C9—C8—H8	125.5	C27—C28A—H28E	109.0
C7—C8—H8	126.3	H28D—C28A—H28E	109.5
C8—C9—N2	106.9 (2)	C27—C28A—H28F	108.9
C8—C9—C10	132.6 (2)	H28D—C28A—H28F	109.5
N2—C9—C10	120.3 (2)	H28E—C28A—H28F	109.5
C11—C10—C9	110.98 (19)	C15—C29—H29A	109.1
C11—C10—C23	110.0 (2)	C15—C29—H29B	110.3
C9—C10—C23	110.00 (18)	H29A—C29—H29B	109.5
C11—C10—C24	112.22 (18)	C15—C29—H29C	109.0
C9—C10—C24	105.9 (2)	H29A—C29—H29C	109.5
C23—C10—C24	107.6 (2)	H29B—C29—H29C	109.5
C12—C11—N3	106.0 (2)	C15—C30—H30A	109.5
C12—C11—C10	131.9 (2)	C15—C30—H30B	108.7
N3—C11—C10	122.0 (2)	H30A—C30—H30B	109.5
C11—C12—C13	108.9 (2)	C15—C30—H30C	110.3
C11—C12—H12	124.3	H30A—C30—H30C	109.5
C13—C12—H12	126.8	H30B—C30—H30C	109.5
C14—C13—C12	107.7 (2)	C20—C31—H31A	109.8
C14—C13—H13	125.9	C20—C31—H31B	109.2
C12—C13—H13	126.4	H31A—C31—H31B	109.4
C13—C14—N3	106.5 (2)	C20—C31—H31C	109.5
C13—C14—C15	133.7 (2)	H31A—C31—H31C	109.5
N3—C14—C15	119.8 (2)	H31B—C31—H31C	109.5
C14—C15—C16	109.75 (17)	O33—C32—O34	123.5 (2)
C14—C15—C29	109.79 (19)	O33—C32—C20	125.1 (2)
C16—C15—C29	109.15 (19)	O34—C32—C20	111.3 (2)
C14—C15—C30	109.4 (2)	C32—O34—C35	115.9 (2)
C16—C15—C30	110.53 (19)	C36A—C35—O34	108.3 (5)
C29—C15—C30	108.18 (18)	C36—C35—O34	109.9 (2)
C17—C16—N4	106.2 (2)	C36A—C35—H35C	109.8
C17—C16—C15	132.6 (2)	O34—C35—H35C	110.0
N4—C16—C15	121.2 (2)	C36A—C35—H35D	109.7
C16—C17—C18	108.3 (2)	O34—C35—H35D	110.5
C16—C17—H17	125.6	H35C—C35—H35D	108.6
C18—C17—H17	126.1	C36—C35—H35A	110.0
C19—C18—C17	108.0 (2)	O34—C35—H35A	109.8
C19—C18—H18	125.3	C36—C35—H35B	109.0
C17—C18—H18	126.7	O34—C35—H35B	109.5
C18—C19—N4	106.4 (2)	H35A—C35—H35B	108.6
C18—C19—C20	131.2 (2)	C35—C36—H36A	110.0
N4—C19—C20	122.40 (19)	C35—C36—H36B	108.9
C19—C20—C1	111.99 (17)	H36A—C36—H36B	109.5
C19—C20—C31	109.46 (18)	C35—C36—H36C	109.5
C1—C20—C31	109.13 (17)	H36A—C36—H36C	109.5
C19—C20—C32	109.68 (18)	H36B—C36—H36C	109.5
C1—C20—C32	104.12 (17)	C35—C36A—H36D	110.6
C31—C20—C32	112.4 (2)	H36C—C36A—H36D	101.6
C5—C21—H21A	109.3	C35—C36A—H36E	108.9
C5—C21—H21B	110.2	H36D—C36A—H36E	109.5
H21A—C21—H21B	109.5	C35—C36A—H36F	108.8
C5—C21—H21C	108.9	H36D—C36A—H36F	109.5
H21A—C21—H21C	109.5	H36E—C36A—H36F	109.5
H21B—C21—H21C	109.5		
			
C4—N1—C1—C2	−0.6 (3)	N3—C14—C15—C16	46.3 (3)
C4—N1—C1—C20	−177.20 (18)	C13—C14—C15—C29	102.7 (3)
N1—C1—C2—C3	0.4 (3)	N3—C14—C15—C29	−73.7 (3)
C20—C1—C2—C3	176.5 (2)	C13—C14—C15—C30	−15.9 (3)
C1—C2—C3—C4	0.0 (3)	N3—C14—C15—C30	167.72 (19)
C2—C3—C4—N1	−0.4 (2)	C19—N4—C16—C17	−0.9 (3)
C2—C3—C4—C5	−176.6 (2)	C19—N4—C16—C15	−179.13 (19)
C1—N1—C4—C3	0.6 (3)	C14—C15—C16—C17	−112.8 (3)
C1—N1—C4—C5	177.35 (19)	C29—C15—C16—C17	7.6 (3)
C3—C4—C5—C6	−146.8 (2)	C30—C15—C16—C17	126.4 (3)
N1—C4—C5—C6	37.4 (3)	C14—C15—C16—N4	64.9 (3)
C3—C4—C5—C21	93.5 (3)	C29—C15—C16—N4	−174.8 (2)
N1—C4—C5—C21	−82.2 (2)	C30—C15—C16—N4	−55.9 (3)
C3—C4—C5—C22	−25.3 (3)	N4—C16—C17—C18	0.5 (3)
N1—C4—C5—C22	158.90 (19)	C15—C16—C17—C18	178.5 (2)
C9—N2—C6—C7	−0.1 (2)	C16—C17—C18—C19	0.0 (3)
C9—N2—C6—C5	−179.93 (19)	C17—C18—C19—N4	−0.5 (3)
C4—C5—C6—C7	−114.5 (3)	C17—C18—C19—C20	179.8 (2)
C21—C5—C6—C7	5.5 (3)	C16—N4—C19—C18	0.9 (3)
C22—C5—C6—C7	125.2 (3)	C16—N4—C19—C20	−179.41 (19)
C4—C5—C6—N2	65.3 (3)	C18—C19—C20—C1	120.4 (3)
C21—C5—C6—N2	−174.73 (19)	N4—C19—C20—C1	−59.2 (3)
C22—C5—C6—N2	−55.0 (3)	C18—C19—C20—C31	−0.7 (3)
N2—C6—C7—C8	−0.4 (2)	N4—C19—C20—C31	179.66 (19)
C5—C6—C7—C8	179.4 (2)	C18—C19—C20—C32	−124.5 (3)
C6—C7—C8—C9	0.8 (3)	N4—C19—C20—C32	55.9 (3)
C7—C8—C9—N2	−0.8 (3)	C2—C1—C20—C19	137.3 (2)
C7—C8—C9—C10	174.5 (2)	N1—C1—C20—C19	−47.1 (3)
C6—N2—C9—C8	0.6 (2)	C2—C1—C20—C31	−101.4 (3)
C6—N2—C9—C10	−175.44 (19)	N1—C1—C20—C31	74.3 (3)
C8—C9—C10—C11	125.4 (3)	C2—C1—C20—C32	18.9 (3)
N2—C9—C10—C11	−59.8 (3)	N1—C1—C20—C32	−165.5 (2)
C8—C9—C10—C23	−112.7 (3)	C11—C10—C24—O25	138.4 (3)
N2—C9—C10—C23	62.2 (3)	C9—C10—C24—O25	−100.4 (3)
C8—C9—C10—C24	3.3 (3)	C23—C10—C24—O25	17.2 (3)
N2—C9—C10—C24	178.14 (19)	C11—C10—C24—O26	−44.0 (3)
C14—N3—C11—C12	0.4 (2)	C9—C10—C24—O26	77.3 (2)
C14—N3—C11—C10	177.46 (19)	C23—C10—C24—O26	−165.12 (19)
C9—C10—C11—C12	126.1 (3)	O25—C24—O26—C27	−3.1 (4)
C23—C10—C11—C12	4.2 (3)	C10—C24—O26—C27	179.1 (2)
C24—C10—C11—C12	−115.5 (3)	C24—O26—C27—C28	88.2 (4)
C9—C10—C11—N3	−50.1 (3)	C24—O26—C27—C28A	165.5 (6)
C23—C10—C11—N3	−172.0 (2)	C19—C20—C32—O33	−37.8 (3)
C24—C10—C11—N3	68.3 (3)	C1—C20—C32—O33	82.2 (3)
N3—C11—C12—C13	−0.3 (3)	C31—C20—C32—O33	−159.8 (2)
C10—C11—C12—C13	−177.0 (2)	C19—C20—C32—O34	145.66 (19)
C11—C12—C13—C14	0.2 (3)	C1—C20—C32—O34	−94.3 (2)
C12—C13—C14—N3	0.1 (2)	C31—C20—C32—O34	23.6 (3)
C12—C13—C14—C15	−176.7 (2)	O33—C32—O34—C35	−5.7 (3)
C11—N3—C14—C13	−0.3 (2)	C20—C32—O34—C35	170.92 (19)
C11—N3—C14—C15	177.00 (18)	C32—O34—C35—C36A	−107.8 (8)
C13—C14—C15—C16	−137.3 (3)	C32—O34—C35—C36	−177.0 (2)

### Hydrogen-bond geometry (Å, °)

**Table:**

*D*—H···*A*	*D*—H	H···*A*	*D*···*A*	*D*—H···*A*
N2—H2N···O25^i^	0.86 (2)	2.37 (2)	3.211 (3)	167 (2)

Symmetry codes: (i) −*x*+3/2, *y*+1/2, −*z*+1/2.
